# Role and Mechanism of the Renin-Angiotensin-Aldosterone System in the Onset and Development of Cardiorenal Syndrome

**DOI:** 10.1155/2022/3239057

**Published:** 2022-01-24

**Authors:** Kexin Ma, Weifang Gao, Huazhou Xu, Wenjie Liang, Guoping Ma

**Affiliations:** ^1^The First Hospital of Hebei Medical University, Shijiazhuang 050000, China; ^2^Hebei Key Laboratory of Integrative Medicine of Liver-Kidney Patterns, Institute of Integrative Medicine, Hebei University of Chinese Medicine, Shijiazhuang, 050200 Hebei, China

## Abstract

Cardiorenal syndrome (CRS), a clinical syndrome involving multiple pathological mechanisms, exhibits high morbidity and mortality. According to the primary activity of the disease, CRS can be divided into cardiorenal syndrome (type I and type II), renal heart syndrome (type III and type IV), and secondary heart and kidney disease (type V). The renin-angiotensin-aldosterone system (RAAS) is an important humoral regulatory system of the body that exists widely in various tissues and organs. As a compensatory mechanism, the RAAS is typically activated to participate in the regulation of target organ function. RAAS activation plays a key role in the pathogenesis of CRS. The RAAS induces the onset and development of CRS by mediating oxidative stress, uremic toxin overload, and asymmetric dimethylarginine production. Research on the mechanism of RAAS-induced CRS can provide multiple intervention methods that are of great significance for reducing end-stage organ damage and further improving the quality of life of patients with CRS.

## 1. Introduction

The incidence of heart and kidney disease, especially chronic heart failure (CHF) and chronic kidney disease (CKD), is increasing yearly with changes in lifestyle and increases in pressure. CHF is a health problem worldwide, affecting 2% to 3% of adults [1]. CKD is also a global public health problem, and the prevalence of CKD has increased to 8–16% in adults worldwide over the past 2 decades [2]. CHF and chronic renal failure often occur simultaneously or successively. According to statistics, approximately 20% of heart failure (HF) patients have moderate to severe renal dysfunction, and greater than 60% exhibit at least mild renal dysfunction. The incidence of CHF is also 15-fold higher in CKD patients compared with healthy individuals. In addition, the increase in annual mortality related to dialysis is greater than 20%, and half of these deaths are cardiovascular related [3]. Comorbidities of these conditions are an indicator of poor prognosis, prolonged length of stay, and increased morbidity and mortality [4].

Cardiorenal syndrome (CRS) refers to a complex pathophysiological disorder caused by cardiac and renal insufficiency, and these processes interact with each other [5, 6]. CRS is divided into 5 types based on primary disease activity. Type I and type II are referred to as cardiorenal syndrome, type III and type IV are called renal heart syndrome, and type V refers to heart and kidney involvement caused by simultaneous systemic diseases [7]. Homeostasis and hemodynamics in the body are jointly regulated by the interdependence of the heart and kidneys. The heart depends on the kidneys, which monitor the balance of water and salt; conversely, the kidneys rely on the heart, which generates blood flow and pressure [8]. Primary dysfunction of one organ often leads to secondary dysfunction in another organ, resulting in a vicious cycle and eventually leading to cardiorenal syndrome [4]. Excessive activation of the renin-angiotensin-aldosterone system (RAAS) plays a key role in this process [9].

## 2. Brief Introduction of the RAAS

The RAAS is an important humoral regulatory system of the body, comprising enzymes and peptides. The system widely exists in various tissues and organs, such as the myocardium, vascular smooth muscle, and kidney, and participates in the regulation of target organ function [10]. When the renin content in the blood increases, angiotensinogen (Ang) produced by the liver is hydrolyzed into angiotensin I (Ang I), which promotes the secretion of epinephrine and norepinephrine. Under the action of angiotensin-converting enzymes (ACEs) produced by the lungs, Ang I is converted into angiotensin II (Ang II) [11]. Ang II is converted into angiotensin III (Ang III) under the action of aminopeptidase.

Ang II is considered to be the most important effector of the RAAS [12]. Ang II-stimulated AT_1_R regulates several physiological processes, including vasoconstrictive effect, ventricular hypertrophy, myocardial infarction, atherosclerosis, reactive oxygen species (ROS) generation, tissue inflammation, and aldosterone synthesis [10, 12–14]. Ang II-regulated AT_2_R promotes nitric oxide production and counteracts inflammation [15]. It is worth mentioning that activated AT_1_R may facilitate the internalization of ACE_2_ that is a cellular entry receptor for SARS-CoV-2, further stimulating ACE_2_-related internalization of SARS-CoV-2 into affected cells [16, 17]. The adrenal AT_1_R is regulated to promote aldosterone synthesis and secretion through Gq/11 protein and *β*-arrestin signaling [18].

Aldosterone exerts physiological and pathological effects by activating the mineralocorticoid receptor (MR). The activation of MR can induce cardiac, vasculature, and kidney inflammation and fibrosis and increase the susceptibility to atrial and ventricular arrhythmias through sympathoexcitation [12, 19–22]. Aldosterone can increase the production of ROS which induces a loss of dysregulated dynamics and bioenergetics in the organelle and promotes in mitochondrial the production of high energy phosphates [19, 23, 24]. These effects can lead to mitochondrial and adrenergic receptor dysfunction and coronary vasoconstriction which can lead to HF. Moreover, aldosterone stimulates epidermal growth factor receptor activation through activating MR, which regulates the RAAS and ultimately modulates cardiac physiology [24].

Therefore, the RAAS plays a significant role in maintaining homeostasis of cardiovascular function and the balance of electrolytes and bodily fluids and regulating blood pressure, renal blood flow, and urine production [9, 11, 19, 25, 26].

## 3. Regulation of Renin Secretion

**Figure 1:**
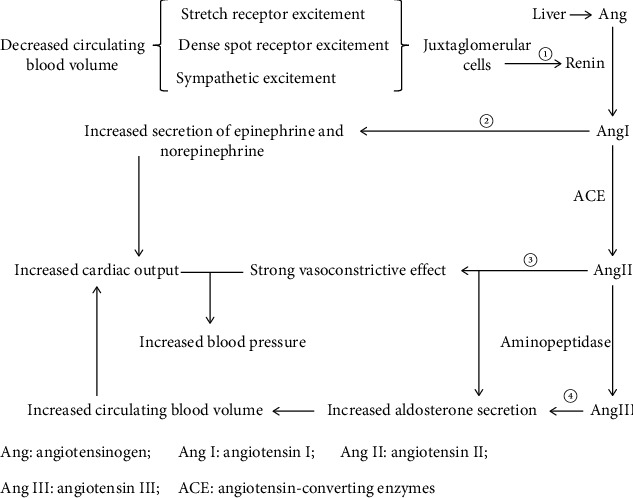
Schematic representation of RAAS activation and its role. RAAS comprises enzymes and peptides. ① Renin secretion is increased under the condition of decreased circulating blood volume. ② Ang I promotes the secretion of norepinephrine and adrenaline, which enhance myocardial contractility and further increase cardiac output. ③ Ang II has a strong vasoconstrictive effect that acts in combination with increased cardiac output to maintain blood pressure stability. ③-④ Both Ang II and Ang III stimulate aldosterone secretion, thereby increasing circulating blood volume.

The process of renin secretion is regulated by the stretch receptors of the vascular wall, dense plaque receptors, and sympathetic nerves [27, 28]. The stretch sensor can sense changes in renal blood flow. When the renal blood flow is decreased, stimulation of the stretch sensor on the wall of the arteriolar arteries is weakened. This information is transmitted to juxtaglomerular cells, which increases the amount of renin excreted in their secreted particles. Dense plaques sense changes in NaCl content in tubule fluid. When renal blood flow is reduced, the Na^+^ content in the tubule fluid is decreased, and the dense spot receptor is further activated. This information is transmitted to periglomerular cells, leading to an increase in renin secretion. When the circulating blood volume is decreased, the sympathetic nervous system (SNS) is activated in a compensatory manner and also promotes renin secretion (Figure 1) [29].

### 3.1. RAAS Activation in CHF

The increases in renal sympathetic nerve activity responsiveness to CHF, which contributes to renin release, further activating RAAS [30, 31]. Under the action of norepinephrine and epinephrine released by Ang I and renal vasoconstriction effect of Ang II, myocardial contractility is enhanced, and peripheral blood vessels are constricted to maintain blood pressure [31] (Figure 1). These effects also result in reduced renal perfusion which contribute to the renal failure [31, 32]. Interestingly, studies of HF in sheep indicate that elevated levels of norepinephrine and Ang II can moderate the renal vascular responses by downregulation of AT1R in the renal medulla and a decrease in renal vascular responsiveness to *α*1-adrenoceptor stimulation, which act to maintain renal function [31].

### 3.2. RAAS Activation in CKD

CKD is a pathological condition associated with an unexplained decrease in the glomerular filtration rate (GFR) (<0.06 L/min) for more than three months. Due to reduced renal blood flow and GFR, the stretch receptors of the arteriolar artery and dense plaques are excited, renin secretion increases, and the RAAS is subsequently activated [27, 28]. Under the combined action of Ang II and Ang III, the stability of renal blood flow and the GFR are maintained [31]. As a result, sodium-water retention and toxin accumulation increase the hemodynamic load.

## 4. The Role of RAAS-Mediated Oxidative Stress in CRS

### 4.1. RAAS-Mediated Oxidative Stress

Excessive activation of RAAS causes abnormal production of Ang II and aldosterone in the blood circulation, which can activate nicotinamide adenine dinucleotide phosphate (NADPH) oxidase in the heart, promoting ROS generation and leading to oxidative stress [11, 24, 33]. Oxidative stress promotes the development of arteriosclerosis and cardiac fibrosis, accelerating the occurrence and development of CRS [34, 35]. The positive feedback mechanism between oxidative stress and RAAS accelerates the process of CRS [36].

### 4.2. The Functions of Oxidative Stress-Induced Arteriosclerosis in CHF and CKD

**Figure 2:**
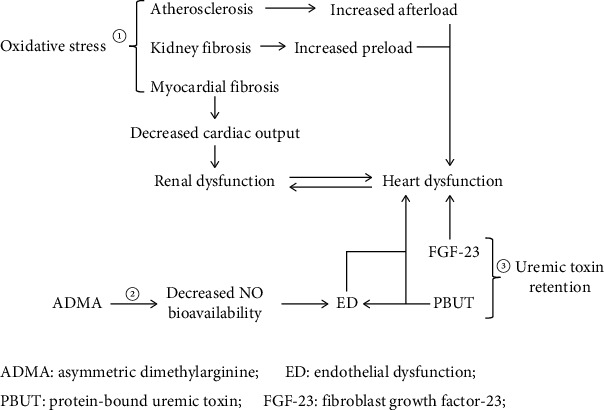
Schematic representation of RAAS-mediated CRS. ① RAAS-mediated oxidative stress not only increases the preload and afterload of the heart by inducing renal fibrosis and atherosclerosis, respectively, but also induces cardiac fibrosis, which further leads to cardiac dysfunction, reduced circulating blood volume, and ultimately renal insufficiency. ② FGF-23 alters the functional activities of the heart by inducing atrial fibrillation, left ventricular hypertrophy, and cardiac fibrosis. PBUT not only causes endothelial dysfunction but also induces cardiac dysfunction by inducing cardiomyocyte fibrosis and apoptosis. ③ RAAS-mediated ADMA production induces endothelial dysfunction by reducing NO production. Endothelial dysfunction increases cardiac preload by reducing the glomerular filtration rate, further leading to cardiac dysfunction.

The biological characteristics of lipids and lipoproteins in the circulating blood are altered by oxidative stress. In human and in vitro studies, oxidized low-density lipoprotein (OX-LDL) enters the vascular endothelium, and the cholesterol it carries accumulates in the arterial wall [37, 38]. Modified LDL stimulates the production of vascular cell adhesion molecules, such as VCAM-1 and P- and E-selectins, on endothelial cells, which results in the recruitment of leukocytes into the subendothelial space. Then, inflammatory cells migrate into the intima [39]. This process is mediated by monocyte chemoattractant proteins. After transformation from monocytes, macrophages express SRA, CD36, LOX-1, and other scavenger receptors, which act through modified LDL internalization. Such cells that harbor lipids are called foam cells, which represent an initial stage of atherosclerotic lesion formation through cytological researches [40, 41]. Oxidative stress in atherogenesis stimulates matrix metalloproteinase release, which leads to a sudden expansion of lesions and promotes arterial thrombosis [42]. Arteriosclerosis aggravates the afterload of the heart and induces compensatory hypertrophy of the heart, further promoting CHF (Figure 2). After the onset of CHF, the circulating blood flow is reduced, and the renal blood flow is decreased, promoting the onset of CKD.

### 4.3. The Effects of Oxidative Stress-Induced Cytokines in CHF and CKD

Oxidative stress causes vascular damage, and the secretion of proinflammatory cytokines, such as interleukin-6 (IL-6) and transforming growth factor-*β* (TGF-*β*), activates fibroblasts and muscle fibroblasts, upregulates collagen production and secretion, and promotes fibrosis of the heart and kidney in rat and human cell studies (Figure 2) [11, 43, 44]. Moreover, human and animal studies have shown that these proinflammatory cytokines subsequently contribute to oxidative stress by increasing NADPH oxidase-generated ROS [45, 46]. Myocardial fibrosis restricts ventricular dilatation, reduces cardiac output, decreases renal blood flow, and causes CKD. Glomerular fibrosis and tubulointerstitial fibrosis lead to decreased renal filtration function, increased fluid retention and preload of the heart, and accelerated cardiac function deterioration [47]. In addition, proinflammatory cytokines aggravate oxidative stress and form a positive feedback loop, eventually leading to a vicious cycle in the study of animals [48].

## 5. The Role of RAAS-Mediated Uremic Toxin Retention in CRS

### 5.1. RAAS-Mediated Uremic Toxin Retention

Uremic toxin refers to metabolic waste that cannot be fully excreted from the body and thus accumulates, causing various symptoms and signs due to a decrease in the number of functional nephrons. Activation of the RAAS causes contraction of the glomerular afferent arterioles, which aggravates renal ischemia and therefore reduces glomerular filtration function. In addition, the decrease in renal blood flow increases the incidence of tubular injury in human study [49]. Both of these effects synergistically cause uremic toxin retention via factors, such as fibroblast growth factor-23 (FGF-23), indocyanine sulfate (IS), and p-cresol sulfate (P-CS).

### 5.2. The Relationship between FGF-23 and CRS

FGF-23 is a bone-derived phosphate-regulating hormone that often occurs in bone metabolic disorders caused by CKD in cat study [50]. FGF-23 promotes the formation of new blood vessels, repairs damaged endothelial cells, and promotes cardiac fibrosis [51]. In human and rat studies, elevated FGF-23 is an independent risk factor for atrial fibrillation (AF), as it can activate protein kinase C and calmodulin-dependent protein kinase II, increase late sodium current and calcium current intensity, and then induce the onset of fatal arrhythmias, such as AF [52, 53]. FGF-23 binds to FGF-23 receptor 4, which activates signal transduction pathways, such as phospholipase C*γ*/calcineurin/NFAT, and participates in the development of left ventricular hypertrophy and cardiac and renal fibrosis in cell, animal, and human studies [51, 54–57]. Animal research shows that FGF23 stimulated the activation of RAAS by increasing the expression of the RAAS-associated genes Agt, Ren, Ace, and Ngal, further resulting in enhanced Ang II and aldosterone synthesis and triggering cardiac hypertrophy and fibrosis (Figure 2) [55]. Therefore, the development of CKD can lead to cardiac insufficiency and further decrease cardiac output, which thus reduces renal blood flow, accelerates the progression of kidney disease, and forms a positive feedback loop to promote the development of CRS.

### 5.3. The Relationship between Protein-Bound Uremic Toxin (PBUT) and CRS

IS and P-CS are PBUTs, and their binding affinity for albumin may be greater than 90% in human studies [58]. After binding to albumin, PBUTs are difficult to clear via glomerular filtration and dialysis. PBUT capacity overload has been identified as a risk factor for heart, kidney, and vascular damage. Cell research shows that PBUTs induce ROS and cause oxidative stress, which affects heart and kidney function [59]. IS participates in the development of mouse myocardial fibrosis by activating nuclear factor-*κ*B (NF-*κ*B) and the transforming growth factor-*β* pathway; P-CS participates in mouse myocardial cell apoptosis [60]. In addition, the physiological functions of endothelial cells and vascular smooth muscle cells are altered by IS and P-CS, affecting their related genes and leading to endothelial dysfunction (Figure 2) [59]. The pathological effects of PBUT reduce the circulating blood volume and further aggravate CRS.

## 6. The Role of RAAS-Mediated Asymmetric Dimethylarginine (ADMA) in CRS

### 6.1. RAAS-Mediated ADMA Generation

ADMA is an endogenous nitric oxide (NO) synthase inhibitor. In CRS, excessive activation of RAAS leads to dysregulation of human dimethylarginine dimethylaminohydrolase (DDAH) and arginine methyltransferase (PRMT), which increase the production of ADMA, affecting CRS evolution by reducing NO bioavailability (Figure 2) [61].

### 6.2. The Effects of ADMA in Endothelial Dysfunction

ADMA inhibits NO production by competing with the substrate I-arginine in human studies [61, 62]. In the human cardiovascular system, NO plays an important role in vascular tone and remodeling and myocardial contractility. NO has several functions, such as regulating hemodynamics, reabsorption of substances, and renin secretion in the kidney [61, 63]. Human myocardial cells isolated from the heart under stress have shown reduced endothelial nitric oxide synthase (eNOS) activity and expression, indicating a decrease in NO. Reduced NO can induce endothelial dysfunction (ED) and oxidative stress [62]. Endothelial injury acts as an initial trigger causing the accumulation and infiltration of multiple-modified low-density lipoprotein particles in the subendothelial space [64]. Oxidative stress aggravates vascular endothelial damage and promotes the onset and development of CHF and CKD, thus promoting CRS development.

ED often leads to lower GFR and diuretic effects, which cause fluid retention and an increased preload [65]. Human and rat studies show that ED can also lead to decreased eNOS expression and thus induce reduced expression of vasodilation factors, such as NO; abnormal endothelium-dependent vasodilation; and the onset of arteriosclerosis and calcification [66, 67]. ED in animal is a key process involved in the development of CRS, and it plays a vital role in the connection between cardiovascular disease and CKD (Figure 2) [68].

## 7. Conclusion

Research on the mechanism of RAAS-induced CRS can provide multiple intervention methods to clinically reduce end-organ damage, thereby ensuring the quality of life of patients with CRS. At present, RAAS inhibitors include angiotensin-converting enzyme inhibitors (ACE-I), angiotensin receptor blockers (ARB), and mineralocorticoid receptor antagonists (MRA). A meta-analysis showed that RAAS blockers are beneficial to alleviate renal function in patients with HF, protect the cardiovascular system from aldosterone-induced pathological remodeling, and reduce the incidence of sudden cardiac death, thereby prolonging survival and reducing mortality [12, 47, 69–71]. Although RAAS inhibitors have significant clinical efficacy, their application is limited due to their limitations.

Finerenone is a nonsteroidal, selective MRA that may have lower risk of hyperkalemia, have more efficient hypotensive effectiveness, and be beneficial to reduce progression of CKD [72]. Large analysis of over 300 000 HF patients points out that compared with RAAS inhibitors, ARB valsartan combined with the neprilysin inhibitor sacubitril is more beneficial to patients with HF and also significantly reduce the rates of GFR decline in CHF, further moderate end-stage kidney disease [13, 72–74]. Also, other therapeutic options include some antidiabetic drugs, for example, sodium-glucose cotransporter 2 (SGLT_2_) inhibitors, dapagliflozin, and canagliflozin that have been shown highly effective in CRS [72, 75–78].

In addition to effective drug to inhibit RAAS, early recognition of RAAS activation is also critical through biomarkers. Studies have showed that the renal resistive index (RRI) may accurately evaluate very early renal damage secondary to CRS as elevated RAAS and may be more sensitive than creatinine in dogs [79–82]. ACE-I and MRA (spironolactone) can be administered early to improve renal function and delay the progression of CRS by monitoring RRI indicators [81]. Urinary angiotensinogen (U-AGT) has been considered not only a biomarker of intrarenal RAAS modulation but also an important biomarker for risk stratification in HF [36, 83, 84]. Studies of patients with renal function impairment showed that U-AGT has a significant positive correlation with urinary hydrogen peroxide (U-H_2_O_2_) that can reflect both systemic and renal oxidative stress [36, 85]. Large body of evidence indicates that the combination of RAAS blockers with vitamin D and/or antioxidants might be useful to reduce RAAS activation and oxidative stress and prevent further cardiac and renal deterioration in CRS through detecting U-AGT and U-H_2_O_2_ [36, 86, 87].
